# The SmartTarget Biopsy Trial: A Prospective, Within-person Randomised, Blinded Trial Comparing the Accuracy of Visual-registration and Magnetic Resonance Imaging/Ultrasound Image-fusion Targeted Biopsies for Prostate Cancer Risk Stratification

**DOI:** 10.1016/j.eururo.2018.08.007

**Published:** 2019-05

**Authors:** Sami Hamid, Ian A. Donaldson, Yipeng Hu, Rachael Rodell, Barbara Villarini, Ester Bonmati, Pamela Tranter, Shonit Punwani, Harbir S. Sidhu, Sarah Willis, Jan van der Meulen, David Hawkes, Neil McCartan, Ingrid Potyka, Norman R. Williams, Chris Brew-Graves, Alex Freeman, Caroline M. Moore, Dean Barratt, Mark Emberton, Hashim U. Ahmed

**Affiliations:** a Research Department of Urology, Division of Surgery and Interventional Science, Faculty of Medicine, University College London, London, UK; b Department of Urology, UCLH NHS Foundation Trust, London, UK; c UCL Centre for Medical Image Computing, Department of Medical Physics & Biomedical Engineering, University College London, London, UK; d Translational Research Office, School of Life and Medical Sciences, University College London, London, UK; e Department of Radiology, UCLH NHS Foundation Trust, London, UK; f Centre for Medical Imaging, Division of Medicine, Faculty of Medicine, University College London, London, UK; g Department of Health Services Research and Policy, London School of Hygiene & Tropical Medicine, London, UK; h Surgical and Interventional Trials Unit, Division of Surgery and Interventional Science, Faculty of Medical Sciences, University College London, London, UK; i Department of Pathology, UCLH NHS Foundation Trust, London, UK; j Division of Surgery, Department of Surgery and Cancer, Faculty of Medicine, Imperial College London, London, UK; k Imperial Urology, Imperial College Healthcare NHS Trust, London, UK

**Keywords:** Biopsy, Diagnostic imaging, Prostatic neoplasms

## Abstract

**Background:**

Multiparametric magnetic resonance imaging (mpMRI)-targeted prostate biopsies can improve detection of clinically significant prostate cancer and decrease the overdetection of insignificant cancers. It is unknown whether visual-registration targeting is sufficient or augmentation with image-fusion software is needed.

**Objective:**

To assess concordance between the two methods.

**Design, setting, and participants:**

We conducted a blinded, within-person randomised, paired validating clinical trial. From 2014 to 2016, 141 men who had undergone a prior (positive or negative) transrectal ultrasound biopsy and had a discrete lesion on mpMRI (score 3–5) requiring targeted transperineal biopsy were enrolled at a UK academic hospital; 129 underwent both biopsy strategies and completed the study.

**Intervention:**

The order of performing biopsies using visual registration and a computer-assisted MRI/ultrasound image-fusion system (SmartTarget) on each patient was randomised. The equipment was reset between biopsy strategies to mitigate incorporation bias.

**Outcome measurements and statistical analysis:**

The proportion of clinically significant prostate cancer (primary outcome: Gleason pattern ≥3 + 4 = 7, maximum cancer core length ≥4 mm; secondary outcome: Gleason pattern ≥4 + 3 = 7, maximum cancer core length ≥6 mm) detected by each method was compared using McNemar's test of paired proportions.

**Results and limitations:**

The two strategies combined detected 93 clinically significant prostate cancers (72% of the cohort). Each strategy detected 80/93 (86%) of these cancers; each strategy identified 13 cases missed by the other. Three patients experienced adverse events related to biopsy (urinary retention, urinary tract infection, nausea, and vomiting). No difference in urinary symptoms, erectile function, or quality of life between baseline and follow-up (median 10.5 wk) was observed. The key limitations were lack of parallel-group randomisation and a limit on the number of targeted cores.

**Conclusions:**

Visual-registration and image-fusion targeting strategies combined had the highest detection rate for clinically significant cancers. Targeted prostate biopsy should be performed using both strategies together.

**Patient summary:**

We compared two prostate cancer biopsy strategies: visual registration and image fusion. A combination of the two strategies found the most clinically important cancers and should be used together whenever targeted biopsy is being performed.

## Take Home Message

Image-fusion results in a clinically significant prostate cancer detection rate were similar to those of visual registration performed by an experienced operator. Detection could be improved by 14% with no adverse effect on patient safety by adding image fusion to conventional visual-registration targeting.

## 1 Introduction

Current management strategies for prostate cancer (PC) depend heavily on appropriate risk stratification, for which adequate tumour sampling and localisation are pivotal. The standard 12-core systematic transrectal ultrasound (TRUS)-guided prostate biopsy has, however, led to overdiagnosis of indolent cancers in some patients and undersampling in others. Development of multiparametric magnetic resonance imaging (mpMRI) has improved diagnostic sensitivity for clinically significant disease while reducing overdetection of clinically insignificant cancer [1], [2], [3].

Several mpMRI-directed or targeted biopsy methods have been established to improve risk stratification: in-bore targeted biopsies, visual registration (also called cognitive registration, mentally translating mpMRI targets onto real-time ultrasound images), and software-based MRI/ultrasound image-fusion systems overlaying MRI targets onto real-time ultrasound images [3]. None has yet established superiority in a clinical setting. Whether visual-registration targeting is sufficient or whether it needs augmentation with image-fusion software has been debated [4]. SmartTarget Biopsy trial (ClinicalTrials.gov NCT02341677) was conducted to compare visual registration with image fusion using a validated [5] MRI/ultrasound fusion system developed in our institution (SmartTarget; technical details included in the Supplementary material).

## 2 Patients and methods

### 2.1 Study design and participants

SmartTarget Biopsy was a prospective, blinded, within-person randomised, paired, validating clinical trial designed in accordance with the IDEAL Collaboration recommendations for medical device evaluation [6]. The study was approved by the London-Dulwich Research Ethics Committee (REF 14/LO/0830) and conducted at University College London Hospital (UCLH). Men with previous mpMRI and TRUS-guided prostate biopsy with or without a PC diagnosis, referred to UCLH for repeat biopsy for accurate diagnosis and further risk stratification, were screened for the study. Eligibility criteria included a discrete lesion seen on mpMRI with Likert scoring 3, 4, or 5, and no hormone therapy (except 5-alpha reductase inhibitors) within the last 6 mo, previous radiation therapy to the pelvis, PC treatment, or evidence of metastatic or nodal disease outside the prostate. Consecutive eligible patients who provided written consent to the trial were enrolled. The trial was completed in accordance with the protocol.

### 2.2 Intervention and follow-up

Before enrolment in the study, all men underwent mpMRI as standard of care in accordance with the British Society of Urogenital Radiology and European Society of Urogenital Radiology standards [7], [8], [9] in sequences as described in our previous studies [1]. These procedures were reported in both written and pictorial form by an experienced uroradiologist with access to clinical information. Each lesion was scored using a five-point Likert system, which was our centre standard at the time of study start, and has been shown to be valid and equivalent to Prostate Imaging Reporting and Data System (PI-RADS) version 1. Our study commenced before PI-RADS version 2 [10], [11].

Each enrolled patient underwent transperineal prostate biopsy under local anaesthetic with the option of sedation and had up to three biopsy samples, each taken using visual estimation and image fusion. Only one lesion was biopsied (the lesion with the highest Likert score, if more than one lesion was present). Biopsies were directed to the target lesion via a 5-mm transperineal grid. Our ethics committee permitted only three needle deployments for each strategy to comply with our prostate biopsy standard operating procedure. Additional samples were collected as clinically indicated but were not analysed for this study. For both biopsy strategies, the grid coordinates were recorded for each needle deployed; each core was potted individually in chronological sequence, and the sequence was recorded.

A different surgeon performed each of the biopsy strategies. The order of the two biopsy procedures was randomised 1:1 to minimise the impact of any visible signs from the first biopsy on the second surgeon. To minimise incorporation bias (bias based on the knowledge of the comparator test results), the equipment was reset by loosening the stepper/ultrasound jig between the two biopsy strategies, requiring the second operator to appropriately position the probe again. All 14 surgeons were trained in visual-registration targeted biopsy within our longstanding, high-volume programme and on the SmartTarget image-fusion software and platform. Surgeons had access to the images before but not during the procedure. The only information on tumour location during the biopsies was generated by the software for the image-fusion biopsy strategy.

The surgeon performing each procedure was also randomised, based on a binary clinical experience ranking to reduce the effect of experience level as a confounder. The randomisation schedule was generated by the study statistician and blocked in groups of four so that there was a balance in allocation after every 20th patient. The randomisation schedule was kept in the clinical trial unit, concealed from clinical staff at all times. After a check of eligibility criteria, the randomised allocation was provided by telephone and in writing immediately before the procedure. Within-person randomisation (ie, comparison of the two strategies performed on each patient) was used to enable evaluation of the detection rate of each strategy and the effect of combining the two.

Biopsy samples were evaluated by a specialist uropathologist with more than 15 yr of experience and classified according to our validated system (Supplementary Table 1) [12]. Each patient completed established, validated instruments for the detection of change in genitourinary function, the International Prostate Symptom Score (IPSS) and International Index of Erectile Function—15 questions (IIEF-15) [13], [14], and the EuroQol—5 Domains—5 Levels (EQ-5D-5L) questionnaire before the procedure, and at 1 and 6 wk after the procedure. Adverse events (AEs) were recorded until 6 wk after the procedure, and serious AEs (SAEs) were reported for 90 d after the procedure.

### 2.3 Outcomes

The prespecified primary outcome was the proportion of men with UCL definition 2 clinically significant disease [15] based on image-fusion biopsy versus that based on visual registration. The primary outcome was therefore Gleason pattern of ≥3 + 4 = 7 or cancer core length of ≥4 mm in any core. The prespecified secondary outcome was the proportion of men with UCL definition 1 clinically significant disease detected by image-fusion biopsy versus visual-registration biopsy, that is, Gleason pattern of ≥4 + 3 = 7 or cancer core length of ≥6 mm in any core. Questionnaires and AEs were used to evaluate the quality of life and safety related to performance of both targeting strategies.

### 2.4 Statistical analysis

Our null hypothesis was that the accuracy of these two biopsy strategies is equivalent, that is, all result pairs are either both positive (a) or both negative (d). Discordant biopsy result pairs are represented by b (visual-registration biopsy positive and image-fusion biopsy negative) or c (visual-registration biopsy negative and image-fusion biopsy positive). For the null hypothesis to be true, b/c = 1. To determine the required study sample size, an estimate of the proportions of the discordant pairs (b and c) was required. As the results of the two strategies are not independent, a test of match-paired binary responses was selected for the primary statistical analysis. For McNemar's test of paired proportions using the Miettinen normal approximation with a two-sided significance level of 0.05, a sample size of 80 achieves a power of at least 0.80 when the discordant proportions are 0.05 and 0.20 of the total number.

If it is assumed that in 80% of men results from one of the methods were classified as UCL definition 2 clinically significant cancer, then either method would have to classify <65% of men as having UCL definition 2 clinically significant cancer for there to be sufficient power to detect a significant difference. Therefore, sample size was driven by the number of men with clinically significant cancer present on visual-registration targeted biopsies and not by the total number of men biopsied. A preplanned evaluation of significant disease prevalence in the first 50 patients biopsied as of 23 July 2016 determined that the disease prevalence assumption was too high; only 26 (52%) had evidence of clinically significant disease by visual-registration biopsy. Thus, the sample size was increased to at least 128 men with both biopsy strategies.

Primary and secondary outcome analyses were conducted on a per-patient basis using SAS (v9.4). All patients who underwent biopsies by both strategies were included in the primary and secondary outcome analyses.

## 3 Results

**Fig. 1:**
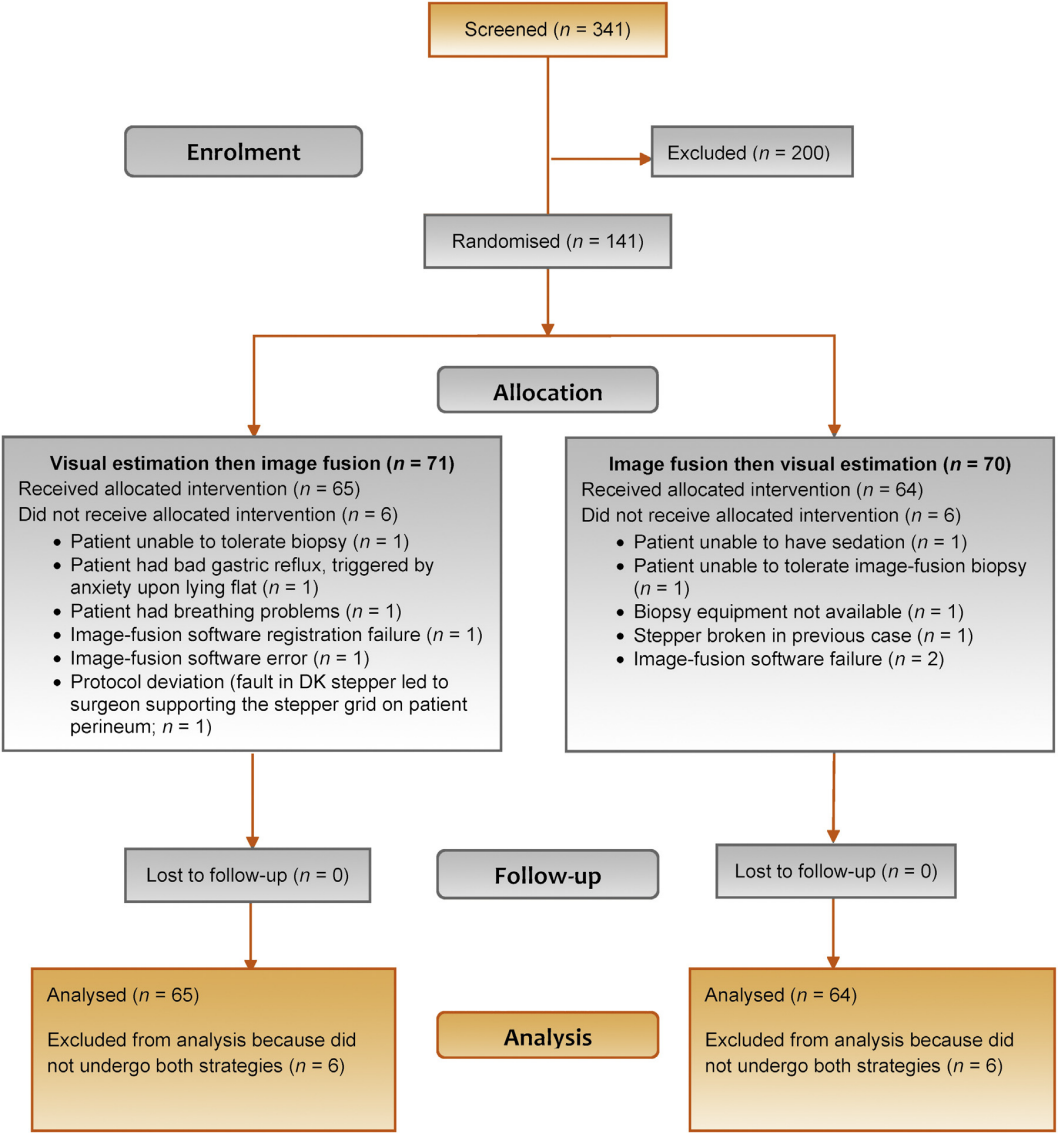
Disposition of patients.

Between 14 November 2014 and 23 September 2016, 341 patients were screened for the study, 141 were enrolled, and 129 underwent both visual-registration and image-fusion biopsies and were analysed for the primary and secondary endpoints. Baseline and demographic characteristics are shown in Table 1. The reasons for randomisation without completion of both biopsies for the other 12 men are provided in Figure 1.

**Table 1:** Demographic and baseline prostate cancer characteristics

Characteristics		*N*	Median (Lower/upper quartile)
Age (yr)		129	65 (58/69)
PSA (ng/ml)		129	8.5 (5.8/11.8)
Ellipsoid lesion volume (cc)^a^		129	0.7 (0.3/1.3)
Prestudy TRUS-guided biopsy total cancer core length (mm)		35^b^	3 (1/6)
**Prestudy TRUS-guided biopsy (*****N*** **=** **95)**^c^			
Gleason pattern, *n* (%)			
3 + 3	55 (58)		
3 + 4	28 (29)		
4 + 3	5 (5)		
Other^d^	5 (5)		
Missing	2 (2)		
**Prestudy multiparametric MRI (*****N*** **=** **129)**			
Likert score, *n* (%)		Target location (base/mid/apex), *n* (%)	
3	22 (17)	Mid	45 (35)
4	67 (52)	Base	27 (21)
5	40 (31)	Apex	19 (15)
**Target location (anterior/posterior),*****n*****(%)**		Mid & apex	18 (14)
Posterior	91 (71)	Base & mid	14 (11)
Anterior	33 (26)	Base & mid & apex	6 (5)
Both	5 (4)		

MRI = magnetic resonance imaging; PSA = prostate-specific antigen; TRUS = transrectal ultrasound.

a Height × length × width × 0.52.

b Men with a prostate cancer diagnosis at study entry for whom the report from prestudy biopsy included information on cancer core length.

c Patients with cancer diagnosis on prestudy TRUS-guided biopsy.

d Benign (2), inflammation (1), no cancer (1), prostatic intraepithelial neoplasia atypical glans (1).

UCL definition 2 PC was detected using both biopsy strategies combined in 93/129 (72%) men (Table 2). Each strategy detected 80/93 (86%; *p *= 1) of these significant cancers with an overall detection rate of 80/129 (62%). Each method identified 13 cancers that the other missed. The combination of the two methods resulted in a 14% (13/93 cases) improvement in the detection of clinically significant PC. Post hoc analysis of this difference showed it to be statistically significant (95% confidence interval: 7.6–22.5). UCL definition 1 PC was detected in 66/129 (51%) men. Of these 66 men, 52 (79%) were identified by visual-registration targeting and 48 (73%) by image-fusion targeting (*p *= 0.5). Image fusion detected 14 definition 1 cancers that visual-registration biopsies missed, and visual registration detected 18 definition 1 cancers that image fusion missed. A post hoc analysis in men with ≥Gleason 3 + 4 = 7 showed a similar pattern. Visual registration detected 71/83 (86%) of these cancers and image fusion 70/83 (84%) with an overall detection rate of 83/129 (64%). The combination of the two methods resulted in a 14% (12/83 cases) improvement in this population. Biopsy characteristics are summarised in Table 3. No differences in patient age, prostate-specific antigen level, total cancer core length, or lesion volume between the concordant and discordant cases was apparent (Supplementary Table 2 and Supplementary Fig. 1–4). An increase in cumulative cancer detection was seen for each additional core taken (Table 4).

**Table 2:** Clinically significant prostate cancer detection

		Visual-registration targeting		
		Negative	Positive	Total
**Definition 2 classification (primary endpoint)**				
Image-fusion targeting	Negative	36	13	49
	Positive	13	67	80
	Total	49	80	129
**Definition 1 classification (secondary endpoint)**				
Image-fusion Targeting	Negative	63	18	81
	Positive	14	34	48
	Total	77	52	129
**Definition 3 classification (Gleason ≥3** **+** **4** **=** **7)**				
Image-fusion targeting	Negative	46	13	59
	Positive	12	58	70
	Total	58	71	129

**Table 3:** Biopsy characteristics

Characteristic	Image-fusion targeting (*N* = 129)	Visual-registration targeting (*N* = 129)
Gleason pattern, *n* (%)		
3 + 3	18 (14)	15 (12)
3 + 4	54 (42)	57 (44)
4 + 3	15 (12)	11 (9)
Not gradable	–	2 (2)
No cancer	40 (31)	42 (33)
Total cancer core length (mm), median (IQR) [*n*]	6 (0–15) [88]	5 (0–13) [90]
Maximum cancer core length (mm), median (IQR) [*n*]	4 (0–6) [129]	4 (0–7) [129]
Risk category, *n* (%)		
Definition 1	48 (37)	52 (40)
Definition 2	80 (62)	80 (62)
Positive cores out of total of 3 per strategy, *n* (%)		
0	40 (31)	42 (32)
1	21 (16)	25 (19)
2	36 (28)	27 (21)
3	42 (33)	34 (26)

IQR = interquartile range.

**Table 4:** Sampling efficiency by number of needle deployments

	Sampling efficiency (%)		
	One	Two	Three
Cancer core length ≥4 mm			
Visual registration	34	46	50
Image fusion	34	46	53
Gleason score ≥(3 + 4)			
Visual registration	36	49	55
Image fusion	40	45	55
Clinically significant disease (definition 2)			
Visual registration	43	57	62
Image fusion	46	55	62

Safety findings after the two biopsy strategies were consistent with the safety profile associated with either strategy performed alone [12], [16]. Three patients experienced AEs related to the biopsy procedure: two patients with events (urinary retention with catheterisation for 1 wk and urinary tract infection) that were mild in severity, and one patient with moderate nausea and vomiting. No SAEs were reported. No statistically significant difference in patient-reported outcome scores (IPSS, IIEF-15, and EQ-5D-5L) was seen between baseline and follow-up (median [interquartile range] of follow-up of 74 [49–105] d; Supplementary Table 3 and Supplementary Fig. 5–7). Individual variation was seen in IIEF-15 scores (overall and domain specific) and in health-related quality-of-life scores measured by EQ-5D-5L (Supplementary Fig. 5–7).

## 4 Discussion

This study, which directly compared transperineal image-fusion and visual-registration biopsy strategies, found no statistically significant difference in overall detection rates of clinically significant PC. Both strategies missed clinically significant cancers detected by the other strategy and so should be used in combination to optimise cancer detection.

The recent publication of the PROMIS trial [1] will increase demand from patients and policymakers to implement an mpMRI-based pathway given the degree of diagnostic superiority that was shown for this method compared with the standard of care. Moreover, the number of studies that have demonstrated increased detection of clinically significant PC using mpMRI targeting (of whatever kind) compared with TRUS-guided biopsy continues to grow [3], [4], [17], [18]. The recent PRECISION randomised controlled trial demonstrated the superiority of MRI-targeted biopsies over systematic biopsies [19]. Omitting the systematic template biopsy and performing only targeted biopsies may have maintained high diagnostic yield for clinically significant cancer in this trial, and reduced patient and healthcare resource burden. However, the optimal method for targeting biopsies has yet to be defined.

The SmartTarget Biopsy study design had a number of important strengths. First, the paired cohort design (both strategies conducted in each patient) allowed both a comparison of detection rates between them and an evaluation of the benefits and risks of combining the two strategies. To minimise potential incorporation bias sources, we randomised the order of the two biopsy strategies for each patient and reset the equipment to a default setting before each biopsy strategy. A double-blind, parallel-group clinical trial would provide confirmation of detection rate similarity afforded for the two strategies, but a design that also assessed the additive value might be more challenging. Second, biopsy conducted by 14 urologists who were considered experienced by our senior assessors in visual-registration biopsy performance meant that the visual-registration strategy was as optimised as possible, providing a robust comparator to image fusion. However, this factor might have provided a comparison level that may, in fact, not represent performance of MRI-targeted biopsies elsewhere by urologists with less experience. Last, the careful prespecified sample size calculation and subsequent increase assured a sample size that minimised type II error, that is, with sufficient power to detect a true difference in PC detection rates between the two biopsy strategies.

Key limitation of this study included capping the biopsy sample number to three per strategy, which may have reduced detection rates for both visual registration and image fusion. This limit may also potentially confound an effect of increasing sample number with the apparent additive effect of the two strategies—the increase in detection rate for the combination of the two strategies. A parallel-group trial, which could maximise the number of needle deployments in an ethically acceptable fashion, could further distinguish the role played by each factor. Furthermore, evaluation of only transperineal biopsy may have limited applicability to transrectal biopsy.

Although our study was conducted in a different population and using different methods, our results are consistent with those of others. Wegelin and colleagues [3] published a systematic review comparing MRI in-bore targeting with a targeted biopsy utilising both visual registration and image fusion. They showed that each method had similar overall cancer detection rates. However, both MRI in bore and image fusion proved to be superior to visual registration for clinically significant cancer detection, although the confidence that we can attribute to the data was limited by the wide variability in detection rates. Among comparative studies performed in expert centres where skill-based biopsy strategies (visual registration) will be an optimal control, most reports suggest that a biopsy using some form of image registration will approximate expert performance [20], [21]. Wysock et al. [21] demonstrated some benefit associated with an image-fusion system for anterior tumours, but that study was conducted using a transrectal approach, which might have made the sampling of these tumours that were furthest away from the needle deployment subject to some systematic error. In contrast, work by Lee and colleagues [22] found that the sampling of transition zone lesions (with the exception of basal lesions) yielded higher cancer detection rates when image-fusion software was used. This difference was attributed to limited registration contouring to the base or difficulty targeting the base on axial views. We found no baseline or imaging parameter that might explain the discordant cases in our study.

Cost is an important consideration but may vary widely depending on both the capital cost of the system and patient volume. A cost-benefit analysis is a complex question beyond this study's scope. However, our results suggest potential benefits of a faster learning curve and higher repeatability that may enable less experienced centres to increase throughput and achieve cancer detection rates equivalent to those of highly experienced centres.

## 5 Conclusions

Visual-registration and image-fusion targeting strategies combined had the highest detection rate for clinically significant cancers. Targeted prostate biopsy should be performed using both strategies together.  

***Author contributions:*** Hashim U. Ahmed had full access to all the data in the study and takes responsibility for the integrity of the data and the accuracy of the data analysis.  

*Study concept and design:* Ahmed, Emberton, Moore, Donaldson, Willis, van der Meulen.

*Acquisition of data:* Donaldson, Hamid, Punwani, Sidhu, Freeman, McCartan, Rodell, Villarini, Bonmati.

*Analysis and interpretation of data:* Hamid, Donaldson, Rodell, Villarini, Bonmati, Hu, Martin, van der Meulen, Williams, Brew-Graves, Emberton, Ahmed.

*Drafting of the manuscript:* Hamid, Emberton, Ahmed.

*Critical revision of the manuscript for important intellectual content:* Donaldson, Hu, Rodell, Villarini, Bonmati, Tranter, Punwani, Sidhu, Willis, van der Meulen, Hawkes, McCartan, Potyka, Williams, Brew-Graves, Freeman, Moore, Barratt.

*Statistical analysis:* Williams.

*Obtaining funding:* Emberton, Moore, Ahmed, Barratt, van der Meulen.

*Administrative, technical, or material support:* Tranter, McCartan, Potyka, Brew-Graves, Rodell, Villarini, Bonmati.

*Supervision:* Ahmed, Emberton, Barratt, Hu.

*Other:* Hu and Barratt are the inventors of the intellectual property associated with the SmartTarget software. Barratt, Hu, Bonmati, Rodell, and Martin designed and built the SmartTarget guidance system used in this study.  

***Financial disclosures:*** Hashim U. Ahmed certifies that all conflicts of interest, including specific financial interests and relationships and affiliations relevant to the subject matter or materials discussed in the manuscript (eg, employment/affiliation, grants or funding, consultancies, honoraria, stock ownership or options, expert testimony, royalties, or patents filed, received, or pending) are the following: Barratt is a shareholder, director, and chief scientific officer for SmartTarget Ltd (a UK-registered spin-out company of the Centre for Medical Image Computing at University College London, which is commercialising the image guidance device used in the submitted work) and is the inventor named on two patents owned by SmartTarget Ltd (one issued and one pending). University College London also received equity in SmartTarget Ltd for the licensing of these patents. Hu is a shareholder in SmartTarget. Rodell is an employee of SmartTarget Ltd. Bonmati has received personal fees for consultancy from SmartTarget Ltd. Hawkes is a scientific advisor to SmartTarget Ltd and is cofounder, director, and shareholder of IXICO Plc. Barratt, Hawkes, Emberton, and Ahmed received grant funding for this project from the Health Innovation Challenge Fund, a partnership between the Wellcome Trust and the UK Department of Health. Emberton also reports receipt of personal fees and nonfinancial support from Exact Imaging; grant funding, personal fees, and nonfinancial support from Profound Medical Inc. and SonaCare Medical Inc.; grant funding, nonfinancial support, and other funding from Sophiris Bio Inc.; and grant funding, personal fees, nonfinancial support, and other funding from Steba Biotech SA. Ahmed also reports receipt of grant funding and personal fees from SonaCare Medical Inc. and Sophiris Bio Inc., and grant funding from Trod Medical, Prostate Cancer UK, the Wellcome Trust, the MRC (UK), and NHS England. Punwani reports receiving personal fees from Sophiris Bio Inc. Moore receives funding from Prostate Cancer UK, the Movember Foundation, Institute for Cancer Vaccines and Immunology, and the National Institute for Health Research (NIHR) for research in PC diagnosis, active surveillance, and treatment outcomes. Hawkes, Punwani, and Emberton receive research support from the UK's NIHR UCLH/UCL Biomedical Research Centre, and Ahmed receives research support from the UK's NIHR Imperial Biomedical Research Centre. Hawkes and Emberton are NIHR senior investigators.  

***Funding/Support and role of the sponsor:*** This study was funded by the Health Innovation Challenge Fund, Wellcome Trust, and UK Department of Health. The funders of the study had no role in study design, data collection, data analysis, data interpretation, or writing of the report.  

***Acknowledgements:*** We thank the patients who agreed to take part in this study. We thank Anne McDonough, a professional medical writer funded by the UCLH NHS Foundation Trust, for assistance with preparation of this manuscript. This publication presents independent research commissioned by the Health Innovation Challenge Fund (HICF-T4-310), a parallel funding partnership between the Wellcome Trust and the Department of Health. The views expressed in this publication are those of the authors and not necessarily those of the Wellcome Trust or the Department of Health.
