# Supplementary material for: The SmartTarget Biopsy Trial: A Prospective, Within-person Randomised, Blinded Trial Comparing the Accuracy of Visual-registration and Magnetic Resonance Imaging/Ultrasound Image-fusion Targeted Biopsies for Prostate Cancer Risk Stratification
Source: Eur Urol. 2019 May;75(5):733–40. doi: 10.1016/j.eururo.2018.08.007 (PMC6469539; doi:10.1016/j.eururo.2018.08.007)
Supplement: Supplementary file 1 [file mmc1.docx]

**Supplementary material**

**Technical Details of SmartTarget Biopsy Imaging**

The SmartTarget MRI/ultrasound image-fusion system used in this study was a prototype developed by the UCL Centre for Medical Image Computing that has subsequently been further developed to a product that has received both CE certification and US Food and Drug Administration 510(k) approval for prostate biopsy. The first prototype had previously been tested in 160 patients in the PICTURE trial conducted at our institution.^1^The performance of this system used in this study (same system set-up) was fully validated using a prostate imaging phantom and a range of error metrics.^2^ The results show a mean (standard deviation) MRI-ultrasoundimage alignment error of 2.0 (1.0) mm and an overall needle targeting error of 3.0 (1.2) mm using a standard set-up for transperineal prostate biopsy with a 5-mm template grid. It should be noted that the use of a fixed grid limits the accuracy of needle placement when using the system. Therefore, the accuracy may be improved by using an adjustable needle guide or finer grid spacing.

The device is compatible with all magnetic resonance images in standard DICOM format and multiple ultrasound platforms. In this study the prostate and lesion were contoured on T2-weighted images with all sequences used to determine lesion contour. The software deploys advanced machine learning algorithms to compensate for prostate deformation from TRUS probe pressure.^3,4,5^Software engineers provided training and technical support. An engineer was present during some procedures as an observer to record SmartTarget system operational details as part of a separate clinical usability investigation of clinical usability and provided technical assistance only if requested by a clinical operator.

SmartTarget represents the spatial relationship between the lesion and the prostate by a 3D computer model based on prostate/lesion boundary contours manually defined in multiparametric magnetic resonance imaging (MRI) images by urologists not involved in the biopsy procedure. This task is performed using the OsiriX MD software (Pixmeo SARL; Geneva, Switzerland) and an in-house plug-in for OsiriX to export the data. The prostate and lesion contours are transformed into a deformable 3D computer model that can accommodate shape changes due to pressure exerted by a transrectal ultrasound probe and balloon. The urologist undertaking the image-fusion biopsy acquired prostate ultrasound images during the procedure using a biplanar transrectal transducer (Preirus, Hitachi Ltd; Tokyo, Japan) mounted to a fixed electronic digital stepper (DK Technologies GmbH;Barum, Germany).

After acquiring an ultrasound volume, either by translating or rotating the ultrasound probe as ultrasound images were captured by the SmartTarget software, the operating urologist manually defined points on the boundary of the prostate in a selection of captured ultrasound images. The protocol for this step was to define at least six boundary points where prostate capsule is clearly distinguished on at least three ultrasound slices chosen at the discretion of the operator; unlike similar fusion systems, a complete contouring of the capsule in captured ultrasound images is not necessary. Once defined, the deformable 3D model of the prostate and target lesion obtained from the MRI fused automatically with the ultrasound images by fitting the model to the ultrasound points. The accuracy of the alignment of the model and prostate depicted in each slice of the acquired ultrasound volume was then inspected visually and additional ultrasound boundary points added as required. Once an accurate fusion had been achieved, cross-sectional graphical representations of the prostate and lesion model were overlaid on live ultrasound images obtained by the ultrasound scanner. The SmartTarget software calculated the three optimal grid points for each needle deployment based on the criteria of maximising the total predicted lesion tissue core length (equal to the total cancer core length in the case of a biopsy positive for clinically significant cancer).The operator carrying out the image-fusion targeted biopsies was free to target other areas if they felt this was necessary and appropriate.

All the surgeons who participated in the Smart Target BIOPSY study were Urology Fellows with ≥6 months’ training in transperineal targeted biopsies. All had been assessed and approved as independently competent. Each had performed approximately 50 or more procedures and most 100 to 200 procedures.They had also received simulator training in the SmartTarget system and on-site technical support during the procedures

1. Simmons LAM, Kanthabalan A, Arya M, Briggs T, Barratt D, Charman SC, et al. The PICTURE study: Diagnostic accuracy of multiparametric MRI in men requiring a repeat prostate biopsy. Br J Cancer. 2017;116:1159–65.
2. Bonmati E, Hu Y, Villarini B, et al. Technical note: Error metrics for estimating the accuracy of needle/instrument placement during transperineal magnetic resonance/ultrasound-guided prostate interventions. Med Phys. 2018;45:1408–1414.
3. Hu Y, Ahmed HU, Taylor Z, et al. MR to ultrasound registration for image-guided prostate interventions. Med Image Anal. 2012;16:687–703.
4. Hu Y, Gibson E, Ahmed HU, Moore CM, Emberton M, Barratt DC. Population-based prediction of subject-specific prostate deformation for MR-to-ultrasound image registration. Med Image Anal. 2015;26:332–44 .
5. Hu Y, Carter T, Ahmed HU, et al. Modelling prostate motion for data fusion during image-guided interventions. IEEE Trans Med Imaging. 2011;30:1887–900.

**Supplementary Table 1. University College London Hospital classification of prostate cancer**

|  | **Classification** | | |
| --- | --- | --- | --- |
|  | **Definition 1** | **Definition 2** | **Insignificant by Definition 2** |
| Maximum cancer core length (mm) | ≥6 | ≥4 | ≤3 |
| Gleason pattern | ≥4+3 | ≥3+4 | ≤3+3 |
| Interpretation | Lesion with ≥0.5cc volume and presence of dominant Gleason pattern 4 | Lesion with ≥0.2cc volume and any Gleason pattern 4 | Consistent with very low risk cancers |

**Supplementary Table 2.Concordant and discordant results for the primary endpoint**

|  |  | **Visual-registration Targeting** | | |
| --- | --- | --- | --- | --- |
|  |  | **Negative** | **Positive** | **Total** |
| **Image-fusion Targeting** | **Negative** | 36  Concordant negative | 13  Discordant | 49 |
|  | **Positive** | 13  Discordant | 67  Concordant positive | 80 |
|  | **Total** | 49 | 80 | 129 |
|  | | | | |
|  | **Frequency (%)** | | **95% Confidence Interval** | |
| Concordant negative | 36/129 (28) | | 20.4% – 36.5% | |
| Concordant positive | 67/129 (52) | | 43.0% – 60.8% | |
| Discordant | 26/129 (20) | | 13.6% – 28.1% | |

**Supplementary Table 3. Patient-reported outcomes**

| **Patient-reported Outcome Measure** | **Scores**  **Median (Interquartile Range) [N]** | |
| --- | --- | --- |
|  | **Baseline** | **Follow-up** |
| International Prostate Symptom Score | | |
| Total | 11 (6-17) [107] | 11 (6-17) [75] |
| Quality of life | 1.5 (1-2) [110] | 2 (1-2) [75] |
| International Index of Erectile Function – 15 Questions | | |
| Total | 61 (54-67) [71] | 59 (52-68) [35] |
| Erectile function domain | 26 (20-29) [74] | 27 (20-29) [58] |
| Orgasmic function domain | 9 (8-10) [94] | 9 (7-10) [75] |
| Sexual desire domain | 6 (4-8) [116] | 6 (4-8) [37] |
| Intercourse satisfaction domain | 11 (9-12) [73] | 11 (9-12) [70] |
| Overall satisfaction domain | 7 (4-9) [108] | 6 (3-8) [56] |
| Question 2 | 4 (3-5) [95] | 4 (3-5) [74] |
| EuroQol – 5 Domains – 5 Levels | | |
| Visual analogue scale | 80 (75-90) [109] | 80 (70-90) [74] |
| Index value | 0.879 (0.768-1) [110] | 0.879 (0.768-1) [74] |

**Supplementary Figure 1. Age at enrolment (years) [median (IQR)] for all men and by concordant positive (Cp), concordant negative (Cn), and discordant (D) groups for the primary endpoint**

All men (n=129)

65 (50 – 69) years

Concordant negative (n=36)

66 (63.5 – 69.5) years

Concordant positive (n=67)

62 (56 – 68) years

Discordant (n=26)

64.5 (57 – 71) years

**Supplementary Figure 2. Total cancer core length (mm) [median (IQR)] for all men with a positive biopsy and by concordant positive (Cp), concordant negative (Cn), and discordant (D) groups for the primary endpoint**

All men (n=35)

3 (1 – 6) mm

Concordant negative (n=10)

0.5 (0 – 3) mm

Concordant positive (n=18)

4.5 (2 – 15) mm

Discordant (n=7)

4 (2 – 4) mm

**Supplementary Figure 3. PSA in the last 3 months PSA in last 3 months [median (IQR)]for all men with a positive biopsy and by concordant positive (Cp), concordant negative (Cn), and discordant (D) groups for the primary endpoint**

All men (n=124)*

8.45 (5.65 – 11.9) ng/mL

Concordant negative (n=35)

6.9 (5.3 – 9.1) ng/mL

Concordant positive (n=63)

9.2 (5.8 – 14.2) ng/mL

Discordant (n=26)

7.8 (4.5 – 10) ng/mL

**Supplementary Figure 4. MRI lesion ellipsoid volume [median (IQR)] [median (IQR)]for all men with a positive biopsy and by concordant positive (Cp), concordant negative (Cn), and discordant (D) groups for the primary endpoint**

All men (n=114)*

0.7 (0.3 – 1.3) cm^3^

Concordant negative (n=32)

0.7 (0.2 – 1.25) cm^3^

Concordant positive (n=67)

0.8 (0.4 – 1.3) cm^3^

Discordant (n=24)

0.5 (0.2 – 1.1) cm^3^

**Supplementary Figure 5. Distribution of International Prostate Symptom Score by visit**

**Total Score Quality of Life**

**Supplementary Figure 6. Total International Index of Erectile Function – 15 Questions score by visit and change in score for individual patients**

**Supplementary Figure 7. Total EQ-5D-5L index value by visit and changesin index value for individual patients**
